# Determination of two fluoroquinolones and their combinations with hyaluronan effect in *in vitro* canine cartilage explants

**DOI:** 10.7717/peerj.6553

**Published:** 2019-03-12

**Authors:** Puntita Siengdee, Waranee Pradit, Siriwadee Chomdej, Korakot Nganvongpanit

**Affiliations:** 1 Animal Bone and Joint Research Laboratory, Department of Veterinary Biosciences and Public Health, Faculty of Veterinary Medicine, Chiang Mai University, Chiang Mai, Thailand; 2 Excellence Center in Veterinary Bioscience, Chiang Mai University, Chiang Mai, Thailand; 3 Science and Technology Research Institute, Chiang Mai University, Chiang Mai, Thailand; 4 Department of Biology, Faculty of Science, Chiang Mai University, Chiang Mai, Thailand

**Keywords:** Enrofloxacin, Hyaluronan, Cartilage explants, Marbofloxacin, Dog

## Abstract

**Background:**

Previous studies reported the effect of enrofloxacin (Enro) and marbofloxacin (Mar) on cell death and alteration of the key genes involved in catabolic and anabolic processes and demonstrated the beneficial effects of hyaluronan (HA) combined with fluoroquinolones (FQs) on primary canine chondrocytes. This study further determines the effects of these treatments on canine cartilage explants in both normal and interleukin-1 beta (IL-1β)-stimulated conditions.

**Methods:**

We examined sulfate glycosaminoglycan (s-GAG) release, uronic acid (UA) content, and safranin-O staining, as well as the expression patterns of inflammatory, extracellular matrix (ECM) component and enzymes.

**Results:**

Enro treatment alone effectively stimulated proteoglycan anabolism by increasing UA content and glycosaminoglycans (GAGs) in normal and pre-IL-1β-stimulated explant, whereas Mar showed opposite results. The combination of HA and FQs increased s-GAG release and UA content in normal explants in addition to effective down-regulated expression of *MMP3*. HA reduced the adverse effects of Mar by enhancing UA and GAG contents in both normal and pre-IL-1β-explants. Moreover, HA effectively induced *HAS1*and *ACAN*up-regulation and reduced *MMP9, TNF, PTGS2,*and *NFKB1* expression for a long term.

**Discussion:**

Our results suggest the direct effects of Enro and Mar may selectively stimulate the conditioned explants to express MMP-codinggenes and promote gene expression involved in matrix production, pro-inflammatory cytokines, and cell degradation in different directions. HA successfully reduced the adverse effects of FQs by enhancing s-GAG and UA contents and down-regulated expression of MMPs.

## Introduction

On the basis of chronological development, enrofloxacin (Enro: C_19_H_22_FN_3_O_3_) and marbofloxacin (Mar: C_17_H_19_FN_4_O_4_), which have been widely used in veterinary medicine, are second- and third-generation fluoroquinolones (FQs). They commonly contain a fluorine atom typically at the C6 position in their quinoline ring structure, which has a broad antimicrobial activity against aerobic Gram-negative, selected Gram-positive, and mycobacterial pathogens, as well as anaerobes (Appelbaum & Hunter, 2000; Takahashi, Hayakawa & Akimoto, 2003). The mechanism of action of FQs is inhibition of bacterial enzymes, such as DNA gyrase and topoisomerase II, which are necessary for bacterial DNA transcription and replication (Domagala, 1994). FQs have been shown to be effective in the treatment against *Chlamydia* spp., *Staphylococcus aureus*, *E. coli*, *Salmonella* spp., and *mycoplasma* spp., which usually cause septic arthritis (Guardabassi, Jensen & Kruse, 2009). Enro and Mar were approved for veterinary use for dogs in 1998 (Babaahmady & Khosravi, 2011; Sárközy, 2001; Babaahmady & Khosravi, 2011; Pallo-Zimmerman, Byron & Graves, 2010), respectively. With different substituents at the R-1, R-7, and X-8 positions on the quinolone central ring system, they have a large difference in terms of molecular structure, antibacterial activity, spectrum, and pharmacokinetic properties (Andriole, 2005; Domagala, 1994; Farca et al., 2007; Hong, Kim & Kim, 1997; Pallo-Zimmerman, Byron & Graves, 2010; Peterson, 2001; Šeol, 2005). However, improvement of antibacterial activity has been reported to be associated with mammalian cell cytotoxicity (Domagala, 1994). Enro and Mar have been indicated for the treatment of infections, including joint infections, caused by susceptible bacteria in dogs and cats (Pallo-Zimmerman, Byron & Graves, 2010). Joint infection or septic arthritis is a serious condition that can cause significant degenerative joint disease (Shirtliff & Mader, 2002). Enro and Mar are available in both oral and injectable preparations (2003). Oral, intramuscular, and intravenous administrations are common in the treatment of septic arthritis, and intra-articular antibiotic administration is frequently used in equines (Morton, 2005; Schneider et al., 1992) but are quite uncommon in dogs (Hewes & Macintire, 2011). FQs are known to induce adverse effects on articular cartilage, involved with musculoskeletal disorder development, tendinitis, and tendon rupture, particularly in juvenile animals (Akali & Niranjan, 2008;  Committee on Infectious Diseases, 2006; Goldman & Kearns, 2011; Machida et al., 1990). The adverse effects of Enro and Mar have been reported in chondrocytes and tendon cell necrosis and apoptosis, which may be related to tendinopathy and cartilage damage (Hildebrand et al., 1993; Lim et al., 2008). A high dose of Enro treatment (>1,000 µg/mL) leads to inhibition of proteoglycan synthesis in horse articular cartilage (Beluche et al., 1999).

We have recently reported on the cytotoxicity of FQs on primary canine chondrocytes in normal and inflammatory-stimulated explants in combination with HA treatment. Our study has determined the beneficial effects of HA in reducing the adverse effects of Enro treatment at the transcriptional level (Siengdee et al., 2016). The objectives of the current study were to further determine and compare the direct effects of Enro and Mar and examine the beneficial effects of the combination of HA with FQs on normal and interleukin-1 beta (IL-1 *β*)-induced changes in canine articular cartilage explants. We have hypothesized that the effects of Enro and Mar in ECM macromolecules and the expression profile of chondrocytes in the cartilage explants would differ between the treatment groups, and that HA would be able to interact with chondrocytes in the explant through surface receptors in order to reduce the adverse effects of the FQs treatment.

## Materials & Methods

### Canine cartilage explant culture

Normal (no OA lesions) canine articular cartilage samples were prepared from the stifle and carpal joints of canine cadavers (3–5-year-old specimens weighing ∼10–20 kg), which had suffered a car accident and did not show any indication of muscular system disorders or diseases. Cadaver specimens were obtained from the Veterinary Cadaveric Unit, Faculty of Veterinary Medicine, Chiang Mai University, Chiang Mai, Thailand. According to the Animals for Scientific Purposes Act, B.E. 2558 (2015), since a part of this experiment was performed on an animal carcass, no ethical approval was required for this study and this was confirmed by the Animal Ethics Committee, Faculty of Veterinary Medicine, Chiang Mai University (License number U1006312558). The collection of cartilage samples and canine cartilage explant cultures has been previously described before (Siengdee et al., 2016). Briefly, within 6 h after collection, the joints were opened aseptically in the laminar flow hood. Cartilage disks were then sliced from the condyles of the carpal and tarsal joints approximately 6–7 mm in diameter and 1–2 mm in depth. All explants were soaked once with 70% ethanol for 30 s to 1 min and thrice with sterile phosphate-buffered saline, containing 10 × of a routine antibiotic dose (a final concentration of 1,000 units/mL of penicillin, 1,000 µg/mL of streptomycin, and 2.5 µg/mL of amphotericin B). The cartilage disks were weighed, and three disks (total, ∼30–35 mg/well) were placed into the same sterile 24-well plates. The cartilage disks were then incubated in 1 mL of 5% fetal bovine serum (FBS; Gibco, Thermo Fisher Scientific, Waltham, MA, USA) with a routine antibiotic dose (100 units/mL of penicillin, 100 µg/mL of streptomycin, and 0.25 µg/mL of amphotericin B) and were maintained at 37 °C under 5% CO_2_ until the start of the experiment.

### Treatment reagents

Recombinant human IL-1 *β* was obtained from Bio-Techne/R&D Systems (Minneapolis MN, USA) and used at a final concentration of 20 µg/mL. The final concentration of 1.5 mg of medium-molecular-weight HA for intra-articular injection (∼500–730 kDa) was obtained from TRB Chemedica (Bangkok, Thailand). The doses of Mar and Enro were determined based on previous studies *in vitro* both at 200 µg/mL (Lim et al., 2008), and 400 and 1,000 µg/mL of Mar and Enro were used as positive control (Beluche et al., 1999; Peters et al., 2002).

### Experimental design

**Figure 1:**
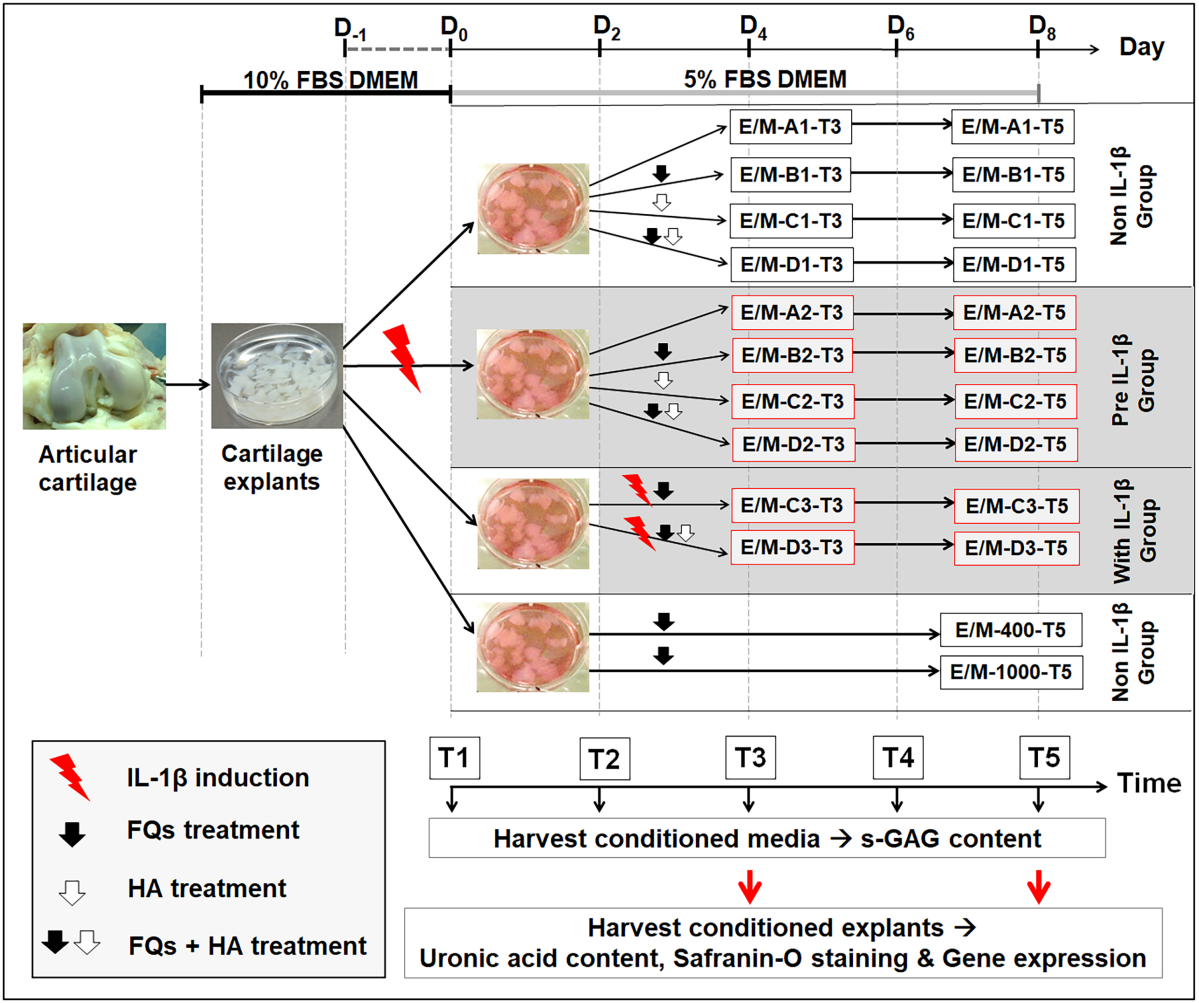
Interrupted time series and experimental design. Cartilage explants were weighed and maintained in 5% FBS growth medium until the experiment started at T1. At T1, one group of the explants (pre-IL-1β) was pre-incubated with 20 µg/mL recombinant human IL-1β in 5% FBS growth medium for 48 h. The non-IL-1β group was replaced with 5% FBS growth medium, comprising the normal cartilage explants. At T2, the sets of explants in the non and pre groups were exposed to FQs with or without HA. The explants in the with IL-1β group were co-treated with IL-1β and FQs with/without HA at the same time. The growth medium was replaced every 2 days (T1–T5) for s-GAG measurement. The conditioned explants were harvested at T3 and T5 for analysis of uronic acid content, safranin-O staining, and gene expression.The explants in the positivFige control groups were treated at 400 or 1,000 µg/mL of each drug, and the conditioned medium and cartilage explants were harvested once at T5.

Figure 1 shows the experimental design of this treatment, and Table 1 presents the codes for the treatment conditions. The canine cartilage explants were divided into three treatment groups under IL-1 *β*-stimulated conditions: (1) non-IL-1 *β* group, cartilage explants exposed to FQs with/without HA; (2) pre-IL-1 *β* group, cartilage explants pre-incubated with recombinant human IL-1 *β* for 48 h to activate cell inflammation before exposure to FQs with/without HA; and (3) with IL-1 *β* group, cartilage explants co-treated with IL-1 *β* and FQs with/without HA at the same time. Treatment groups were inoculated with 5% FBS-supplemented Dulbecco’s modified Eagle’s medium (DMEM) containing 200 ng/mL of Enro or Mar alone and Enro or Mar co-treated with 1.5 mg HA, the negative control for each treatment group was exposed to 5% FBS-supplemented DMEM at equal volume without drugs, and the positive control was treated with 400 and 1,000 µg/mL of each drug through T5 without replacing the growth medium. All cultures were maintained at 37 °C under 5% CO_2_ and examined in three replications. The conditioned medium was collected and replaced every 48 h and stored at −20 °C for further monitoring of sulfate glycosaminoglycan (s-GAG) release. The conditioned cartilage explants, which were assayed for cartilage matrix degeneration assay and examined for histopathology investigation and gene expression, were harvested at days 4 and 8 after treatment (T3 and T5; Fig. 1). Except for the positive control group, the conditioned medium and conditioned explants were collected only once at T5. All cultures were maintained at 37 °C under 5% CO_2_ and examined in three replications.

**Table 1:** Experimental codes of the treatment conditions.

**Enrofloxacin or Marbofloxacin**			
**T3/T5**			
**Code**	**Name**	**Cartilage explant conditions**	**Treatment conditions**
E/M-A1-T3/T5	Non,-FQs	Without IL-1β (normal or negative control)	DMEM with 5% FBS
E/M-B1-T3/T5	non + Enro/Mar	Without IL-1β (normal)	FQs at 200 µg/mL
E/M-C1-T3/T5	non + HA	Without IL-1β (normal)	HA 1.5 mg/mL
E/M-D1-T3/T5	non + HA + Enro/Mar	Without IL-1β (normal)	FQs at 200 µg/mL and HA 1.5 mg/mL
E/M-A2-T3/T5	pre, −FQs	Pre IL-1β-induced	DMEM with 5% FBS
E/M-B2-T3/T5	pre + Enro/Mar	Pre IL-1β-induced	FQs at 200 µg/mL
E/M-C2-T3/T5	pre + HA	Pre IL-1β-induced	HA 1.5 mg/mL
E/M-D2-T3/T5	pre + HA + Enro/Mar	Pre IL-1β-induced	FQs at 200 µg/mL and HA 1.5 mg/mL
E/M-C3-T3/T5	with + Enro	Simultaneous treatment with IL-1β and FQs	FQs at 200 µg/mL
E/M-D3-T3/T5	with + HA + Enro/Mar	Simultaneous treatment with IL-1β and FQs	FQs at 200 µg/mL and HA 1.5 mg/mL
E/M-400-T5E/M-1,000-T5	non + Enro/Mar 400non + Enro/Mar 1,000	Without IL-1β (positive control) Without IL-1β (positive control)	FQs at 400 µg/mL FQs at 1,000 µg/mL

### Determination of s-GAG content

The s-GAG content in the conditioned medium was attributed to the s-GAG accumulation derived from both new s-GAG production and s-GAG degradation from the cartilage disks. The s-GAG content was measured using a colorimetric dye: dimethylmethylene blue (DMMB) binding assay. Subsequently, 16 mg of DMMB was dissolved in 5 mL ethanol, and the volume was adjusted to 1 L with deionized water containing 0.2% sodium formate buffer to adjust the dye solution (pH 3.5) and stored at room temperature in a bottle shielded from light. Chondroitin 6-sulfate from shark cartilage (CS-C; Sigma-Aldrich, St. Louis, MO, USA), which varies from 0 to 60 µg/mL in DMEM, was used as a standard. Subsequently, 50 µL of CS-C or conditioned medium was pipetted into 96-well plates, followed by 200 µL of DMMB dye solution, and the plate was shaken for 5 s. Thereafter, the absorbance was immediately measured at 540 nm using a microplate reader. The serum-free medium was set as blank. The CS-C standard curves were used to calculate the apparent s-GAG levels in each treatment group and calculated as a percentage of s-GAG content relative to the negative control group.

### Quantification of uronic acid

The carbazole colorimetric assay was modified according to the method of Cesaretti et al. (2003) to measure the uronic acid (UA) component in the cartilage matrix. Briefly, treated cartilage disks were digested with 200 µL of papain (2 units) at 60 °C for 48 h. Subsequently, papain cartilage-digested media were diluted with distilled water at 1:5 ratio. Glucuronolactone was used as the standard reagent, and 50 µL of diluted digested sample, or standard, was placed in a 96-well plate, followed by the addition of 200 µL of sodium tetraborate/sulfuric acid reagent (0.025 M Na_2_B_4_O_7_). Thereafter, the 96-well plate was heated at 100 °C for 15 min. After the plate was cooled down to room temperature, 50 mL of carbazole solution was added (0.125% crystallized carbazole in purified absolute ethanol). The sample was heated at 100 °C for 15 min and cooled down to room temperature again. Subsequently, the quantity of UA in the sample was measured using a microplate reader at a wavelength of 540 nm, and the UA content (% µg/mg) was calculated and normalized to the dry weight of each cartilage explant.

### Safranin-O staining of GAGs in the cartilage matrix

The cartilage disks were fixed in 10% neutral buffer formalin fixative solution for approximately 12–24 h at room temperature, embedded in paraffin block, and afterward, the cartilage disks were serially sectioned into approximately 6 µm in depth. The sections of each group were stained with safranin-O to determine the GAG content (Brown, Crawford & Oloyede, 2007; Cook et al., 2010). The cartilage sections were imaged with a Biocom microscope using an AxioCam camera (Carl Zeiss, Oberkochen, Germany) and were performed by processing and calculating the stained image using ImageJ (Király et al., 1996) 1.47 morphometric computer software (Wayne Rasband, National Institute of Health, USA).

### RNA extraction and quantitative real-time PCR (qPCR)

Total RNA was isolated from conditioned explants harvested at T3 and T5 using an innuPREP DNA/RNA Mini Kit (Analytik, Jena, Germany) according to the manufacturer’s instructions. Total RNA was eluted using 20 µL of RNase-free water. The quality and quantity of the RNA samples were checked and measured using a NanoDrop ND-1000 spectrophotometer (Thermo Fisher Scientific, Waltham, MA, USA). Total (DNase-treated) complementary DNA was synthesized using 200 U of Tetro reverse transcriptase enzyme (Bioline, Taunton MA, USA) and 10 mM of oligo (dT) primer in a total reaction volume of 20 µL. The qPCR was performed according to the manufacturer’s recommendations in 10 µL reactions using 2 × SensiFAST SYBR^®^ No-ROX Mix (Bioline) and 20 ng cDNA/reaction well on a LightCycler^®^ 480 (Roche, Basel, Switzerland). qPCR was used to quantify the expression levels of the selected inflammatory, protease, and ECM component genes. Ten pair primer sequences for all genes were designed using *Canis lupus familiaris* cDNA information published on the Ensembl database, and Primer3 software was used for primer design. The sequences of the primer pairs are listed in Table 2. The qPCR reaction was performed at 95 °C for 10 min, followed by 40 cycles for 20 s and 30 s at 95 °C and 60 °C, respectively. All reactions were run in duplicate using RNA isolated from three bio-replicates of the samples. *HPRT1* and *GAPDH* were used as reference control genes. Relative quantification was performed using the 2^−ΔΔ^^*C*^^*T*^ threshold cycle method (Livak & Schmittgen, 2001).

**Table 2:** Sequences of sense and antisense primers used for amplification in qPCR.

**Gene symbol**	**Gene name**	**Ref. Seq. (mRNA)**	**Primer Seq. (5′→3′**)	
***TNF***	Tumor necrosis factor	NM_001003244.4	F	TAGCAAACCCCGAAGCTGAG
			R	TACAACCCATCTGACGGCAC
***PTGS1***	Prostaglandin-endoperoxide synthase 1	NM_001003023	F	GGATGGAGAGATGTAC
			R	CCCAATGAGGATGAGTCGGG
***PTGS2***	Prostaglandin-Endoperoxide Synthase 2	NM_001003354.1	F	GGAGCATAACAGAGTGTGTGATGTG
******			R	AAGTATTAGCCTGCTCGTCTGGAA
***IL-1b***	Interleukin-1, beta	NM_001037971.1	F	GGATGGAAAGCCCACCCTAC
******			R	TCCTGGCCACCTCTGGTATT
***NFKB1***	Nuclear factor of kappa light polypeptide gene enhancer in B-cells 1	NM_001003344.1	F	AAACTGGGCTACTCTGGCAC
******			R	GGCCTCCACCAGCTCTTTTA
***MMP3***	Matrix metallopeptidase 3 (stromelysin 1, progelatinase)	NM_001002967.1	F	GGTTGGAGGTGACAGGGAAG
******			R	CCAGGGAAGGTGGTGAAGTC
***MMP9***	Matrix metallopeptidase 9	NM_001003219.2	F	TTTCGCTATGGCTACACTCAA
******			R	TGCTCCCTAACACCAAACTGA
***TIMP1***	TIMP metallopeptidase inhibitor 1	NM_001003182.1	F	GGACGGACACTTGCAGATCA
******			R	TGCAGGGGATGGATGAACAG
***HAS1***	Hyaluronan synthase 1	XM_005616856.1	F	CAGACACGTGGTCCAAAT
******			R	GCATAGAAGAGCCGCAACAC
***COL2A1***	Collagen, type II, alpha 1	NM_001006951.1	F	CAGCGAGCGTTCCCAAGA
******			R	CAGGCGGAGGAAGGTCAT
***ACAN***	Aggrecan	NM_001113455.1	F	GACCATGTCGTGCAGGTGAC
******			R	ACTGCTCCAGGCGTGTGATG
***HPRT1***	Hypoxanthine phosphoribosyltransferase 1	NM_001003357.1	F	CCTTGGCGTCGTGATTAGTG
******			R	CCGCTCAGTCCTGTCCATAA
***GAPDH***	Glyceraldehyde-3-phosphate dehydrogenase	NM_001003142.1	F	AGTATGATTCTACCCACGGC
			R	CGAAGTGGTCATGGATGACT

### Statistical analysis

Differences between groups were tested by one-way analysis of variance, followed by Tukey’s post-hoc test. Values of *P* < 0.05 were considered statistically significantly different. Pearson correlation coefficient (*r*) tests were used to assess the presence of bivariate correlations between percentages of s-GAG levels of the treatment conditions. In addition, a correlation heat map was generated by hierarchical cluster analysis using MeV version 4.9.0 (Saeed et al., 2006). The software package SPSS 14.0 for Windows (SPSS, Armonk, NY, USA) was used for computations. Data were expressed as the mean ± standard deviation (SD) of separate experiments (*n* = 3, where n represents the number of independent experiments).

## Results

### S-GAG release

s-GAG concentrations in the conditioned medium indicated the effect of FQs on conditioned explants. Here, we calculated the s-GAG concentration as a percentage relative to the negative control group. Our results were significant differences of the percentage of s-GAG levels (*P* ≤ 0.05) in HA treatment groups. The s-GAG levels were decreased in T3, T4, and T5 compared with the T2 and T3, i.e., non + HA (T3–T5), pre + HA (T2–T3 and T2–T5), pre + HA + Enro (T2–T4), pre + HA + Mar (T3–T5), with + HA + Enro (T3–T4), and with + HA + Mar (T2–T5). Pre + Enro was the only group with significant differences between the time period without HA treatment, which was found between T2–T3, T2–T4, and T2–T5 (*P* = 0.019 to *P* = 0.043). The increase in the s-GAG level in the pre-IL-1 *β*-induced group was higher at T2, whereas the increase in the s-GAG level in the with-IL-1 *β*-induced group was higher at T3. Of these, the highest percentage of s-GAG content was found at T3 in the with + HA + Mar group (148.97 ±7.19%), which was also higher than Enro or Mar treated alone in the positive control groups at T5 (Enro 400 = 147.26% ±3.39%, Enro 1,000 = 147.43% ±3.63%, Mar 400 = 148.63% ±4.84%, and Mar 1,000 = 142.64% ±3.15%). However, the s-GAG levels decreased in all treatments after removing IL-1 *β* in both pre- and with-IL-1 *β*-induced explants.

**Figure 2:**
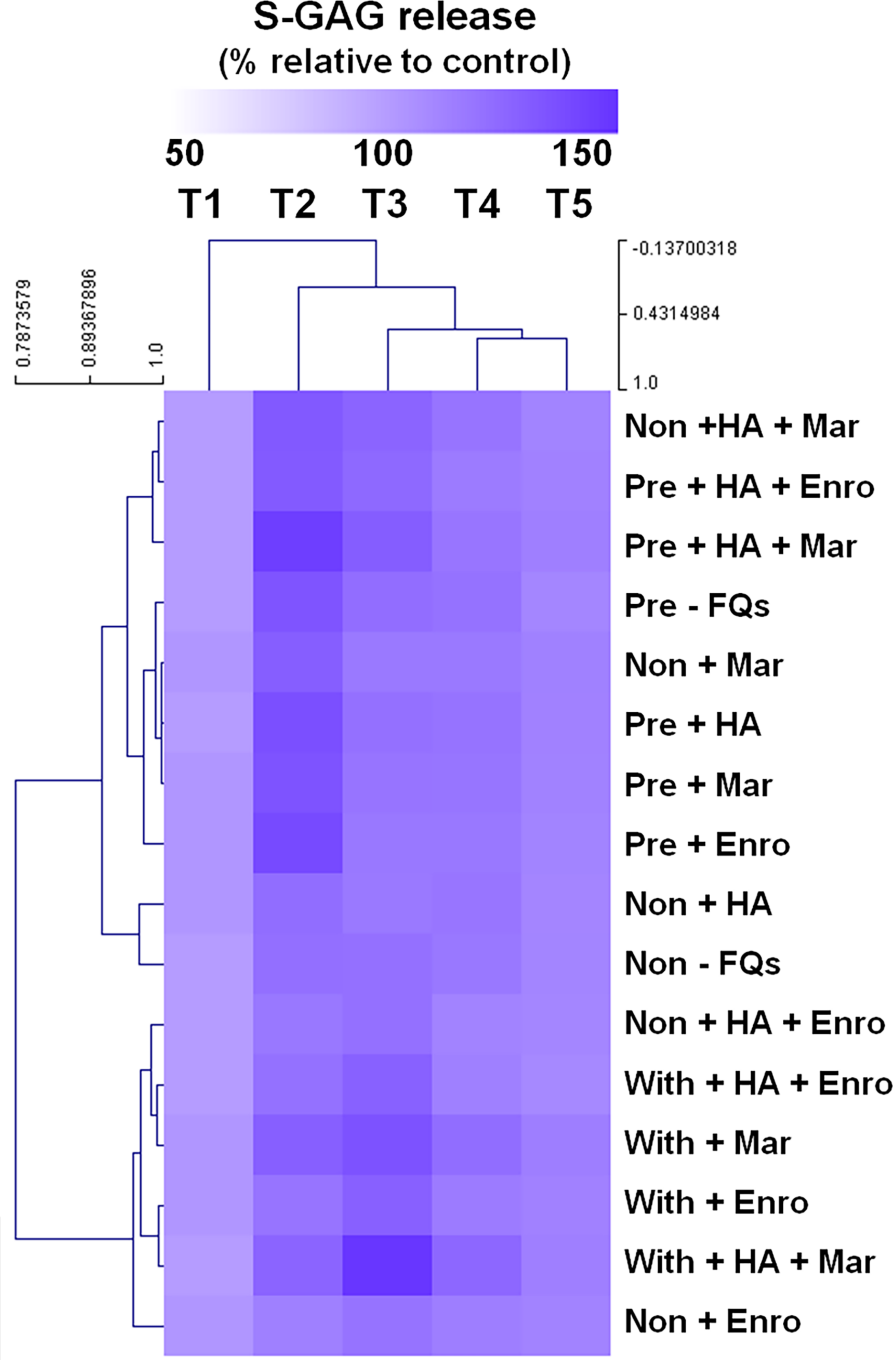
Hierarchical cluster analysis (HCL) summarizing the differences of s-GAG levels. The columns represent the duration from T1 to T5, and each row represents the different treatments. The top of the cluster represent s-GAG levels from 50% to 150% relative to the mean of the negative control. The difference between deeper or light intensityof purple color indicates higher and lower percentages of s-GAG levels. The results display a pattern of thes-GAG levels in different groups of IL-1β-stimulated conditions.

The heat map of the clustered correlation matrix was applied (MeV 4.9.0, Pearson correlation, HCL support tree) to the mean percentages of s-GAG (Fig. 2). The samples were clustered after unsupervised hierarchical clustering. A different pattern of percentages of s-GAG under different IL-1 *β*-stimulated conditions was observed. A high correlation coefficient for s-GAG levels (0.685–0.984, *P* ≤ 0.05) between adjacent clusters of pre-IL-1 *β*-stimulated groups was found to increase from T1 to T2 and increased again at T3–T5, and the negative control group (non, −FQs), non + HA, non + Mar, and non + HA + Mar were also clustered in this pattern. Although the explants treated with IL-1 *β*-stimulated groups were found closely clustered together (*r* = 0.679–0.969, *P* ≤ 0.05), the percentages of s-GAG increased from T1 to T3 and increased again from T4 to T5. The Enro treatments (non + Enro and non + HA + Enro) were clustered in this pattern.

### UA remaining in the explants

**Figure 3:**
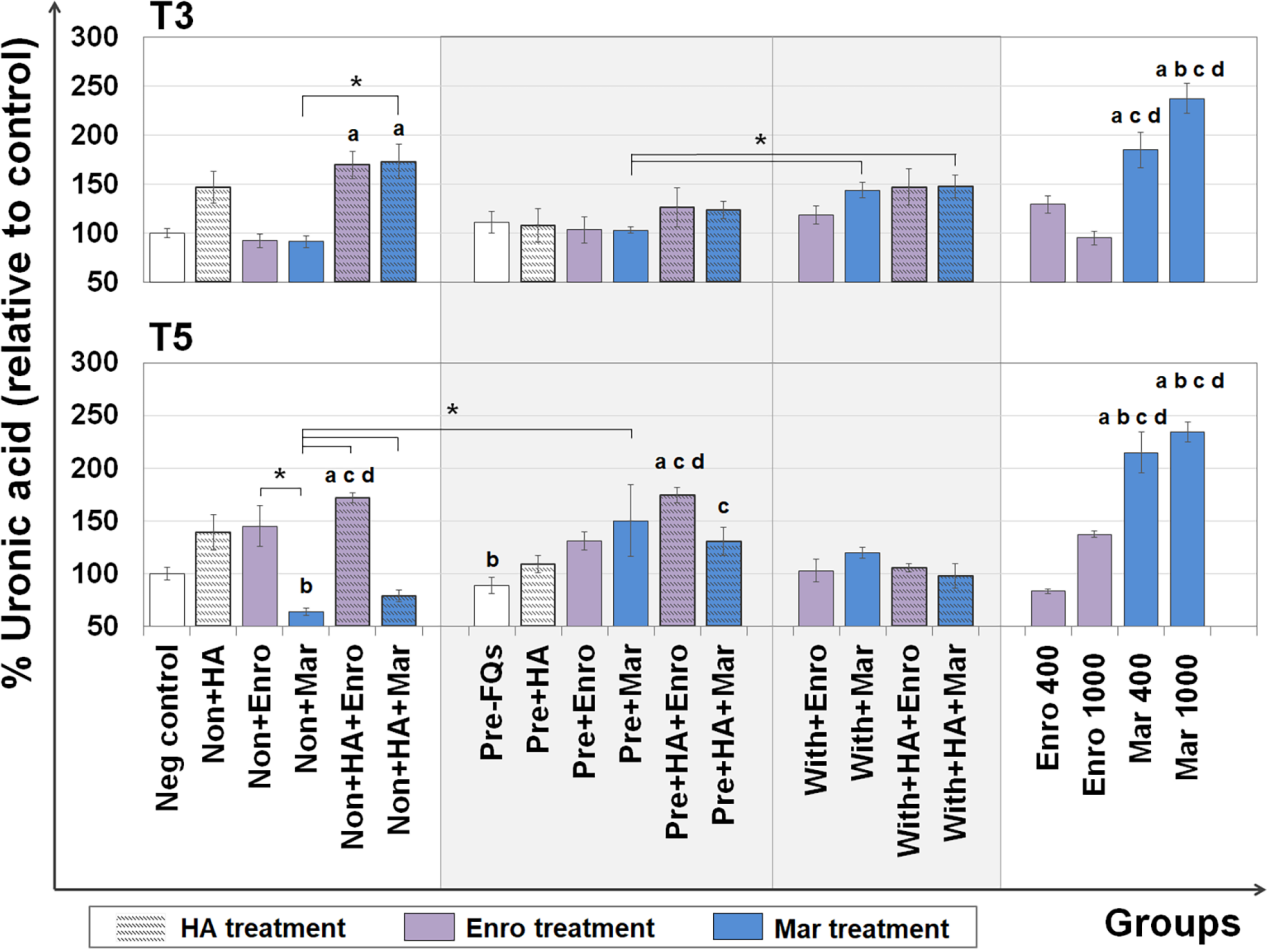
Percentage of uronic acid (UA) content remaining in the explants after FQ treatment with/without HA combination. The UA content of the cartilage explant treatments was calculated and shown in % relative to the negative control group. Significant differences are considered to exist when *P* < 0.05: “a” represents significant differences compared with the negative control group, “b” represents significant differences compared with the non + HA group, “c” represents significant differences compared with the pre-IL-1b induction group (pre, −FQs), “d” represents significant differences compared with the pre + HA group, and the asterisk(*) denotes significant differences between the treatment groups.

The UA content represents the GAG level (excluding keratan sulfate, which contains galactose instead of hexuronic acid) that remains in the explants after treatment. The DMB assay was used to measure the UA content remaining in the cartilage explant disks, and the results are shown in Fig. 3. At T3, the percentages of UA content changes were observed in FQs combined with HA treatment groups, and a slight increase was observed in the single HA-treated groups compared with the negative control. Co-treatment of HA and FQs in normal explants could be attributed to UA accumulation, and non + HA + Enro and non + HA + Mar groups significantly increased the UA content (*P* = 0.034 and 0.019, respectively).

### Safranin-O staining

**Figure 4:**
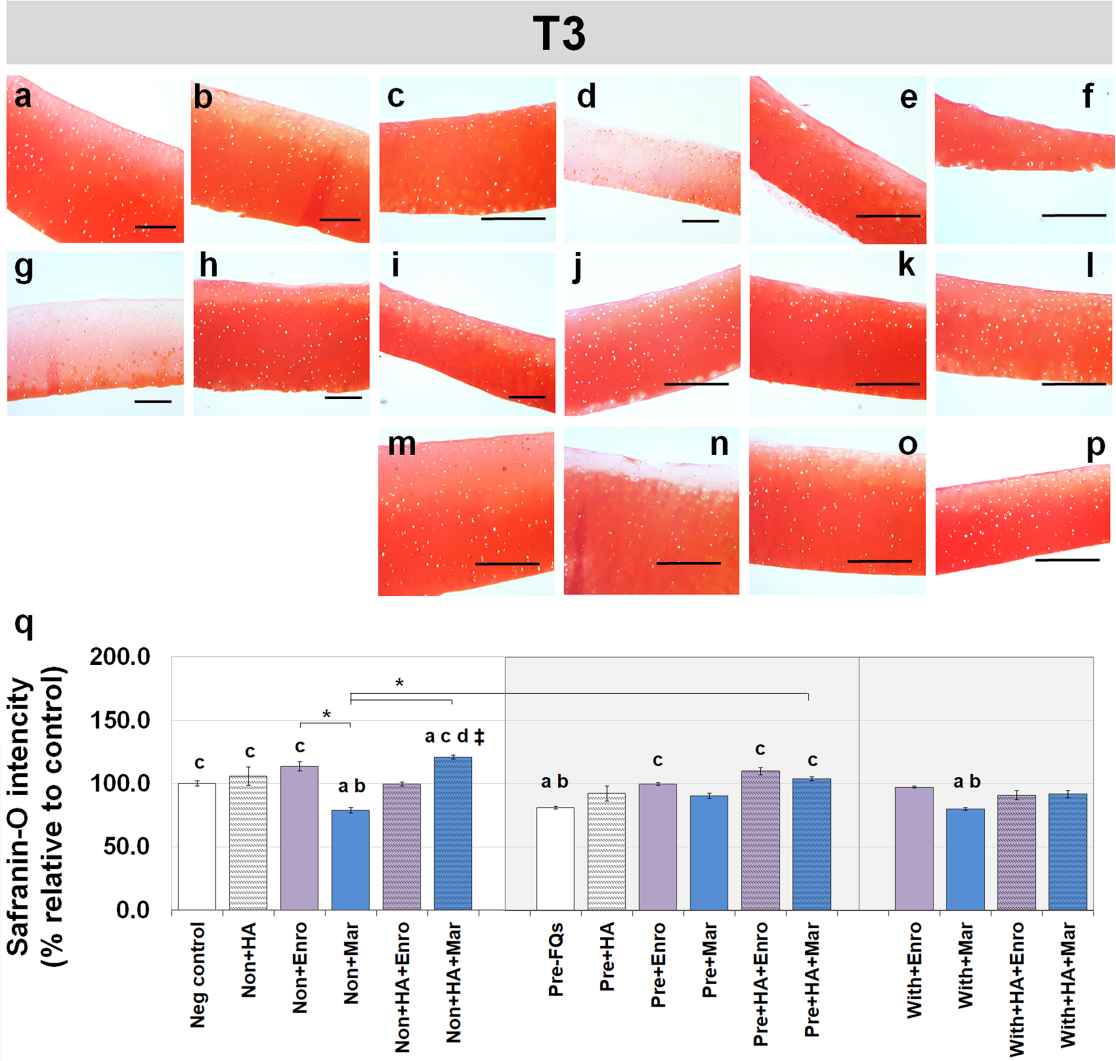
Histological staining with safranin-O (×100) of conditioned canine cartilage explants at T3. The explants were treated with various FQ treatments with or without HA combination. The proteoglycan content in the explant stains is indicated by safranin-O staining. The extent of the morphological change and the proteoglycan content was compared between all treatment groups. Safranin-O staining represents (A) Neg control, (B) non + HA, (C) non + Enro, (D) non + Mar, (E) non + HA + Enro, (F) non + HA + Mar, (G) pre − FQs, (H) pre + HA, (I) pre + Enro, (J) pre + Mar, (K) pre + HA + Enro, (L) pre + HA + Mar, (M) with + Enro, (N) with + Mar, (O) with + HA + Enro, (P) with + HA + Mar”. Scale bar: 100 µm. The safranin-O staining in most Enro treatments was stronger than Mar; however, the highest intensity was found in the non + HA + Mar treatment. HA combined with FQs in both normal and IL-1β-stimulated explant conditions performed rather efficiently, as indicated by the intense red hue. The results of the GAG content (relative to the control) were shown in the bar chart (Q), which is exhibited as the mean ± SD of the triplicate. Significant differences are considered to exist when *P* < 0.05: “a” represents significant differences compared with the negative control group, “b” represents significant differences compared with the non + HA group, “c” represents significant differences compared with the pre-IL-1b induction group (pre, −FQs), “d” represents significant differences compared with the pre + HA group, # represents significant differences compared with the Enro-treated groups, ‡ represents significant differences compared with the Mar-treated groups, and asterisk (*) denotes significant differences between the treatment groups.

**Figure 5:**
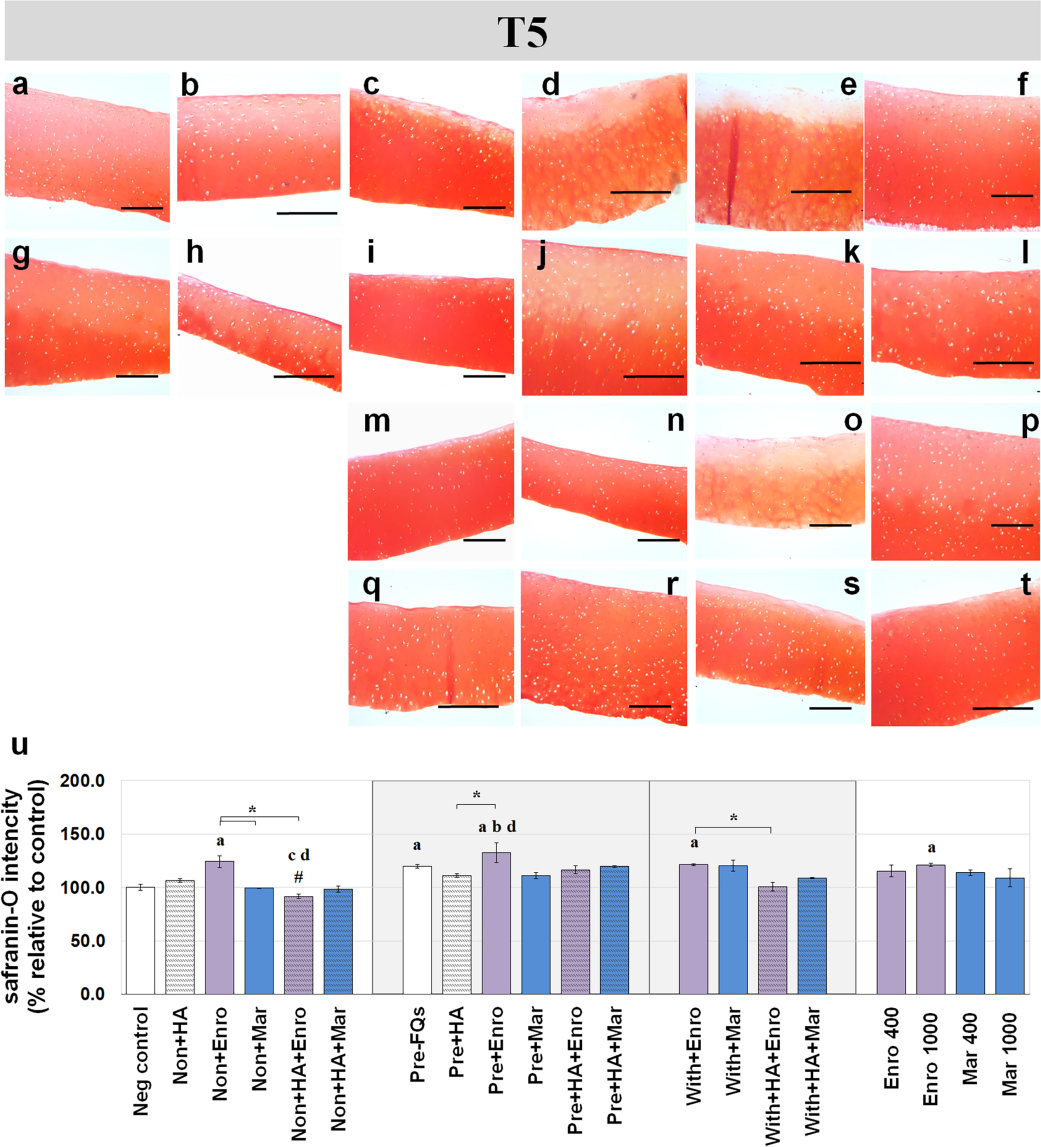
Histological staining with safranin-O (×100) of conditioned canine cartilage explants at T5. The explants were treated with various FQ treatments with or without HA combination. The proteoglycan content in the explant stains is indicated by safranin-O staining. The extent of the morphological change and the proteoglycan content was compared between all treatment groups. Safranin-O staining represents (A) Neg control, (B) non + HA, (C) non + Enro, (D) non + Mar, (E) non + HA + Enro, (F) non + HA + Mar, (G) pre − FQs, (H) pre + HA, (I) pre + Enro, (J) pre + Mar, (K) pre + HA + Enro, (L) pre + HA + Mar, (M) with + Enro, (N) with + Mar, (O) with + HA + Enro, (P) with + HA + Mar, (Q) Enro 400, (R) Mar 400, (S) Enro 1000, and (T) Mar 1000”. Scale bar: 100 µm. The results of the proteoglycan content (relative to the control) were shown in the bar chart (U), which is shown as the mean ± SD of the triplicate. At T5, the safranin-O staining in a single Enro treatment group was stronger than the other groups in every explant condition, particularly in the IL-1β-stimulated explants. The decrease of proteoglycan staining was significantly found in HA combined with Enro treatment in every explant condition, particularly in the non + HA + Enro group. Significant differences are considered when *P* < 0.05: “a” represents significant differences compared with the negative control group, “b” represents significant differences compared with the non + HA group, “c” represents significant differences compared with the pre-IL-1b induction group (pre, −FQs), “d” represents significant differences compared with the pre + HA group, # represents significant differences compared with the Enro-treated groups, ‡ represents significant differences compared with the Mar-treated groups, and the asterisk (*) denotes significant differences between the treatment groups.

Safranin-O staining also identifies the GAG content of the cartilage explants combined with HA shown at different time periods of Enro and Mar treatment in Figs. 4 and 5. We found that the decrease of safranin-O intensity (faded) was more prominent in the superficial area than the deep zone in nearly every treatment group, including the negative control and HA groups at both T3 and T5 (Figs. 4 and 5). At T3, the safranin-O staining in most Enro treatments was stronger than Mar, except when treated on normal explant with HA combination. The highest intensity was found in the non + HA + Mar treatment at 120.72% compared with the negative control group (*P* = 0.022; Fig. 4Q). A significant decrease in safranin-O intensity was observed in the pre, −FQ group and single Mar-treated explants (non + Mar and with + Mar) compared with the negative controls. HA combined with Mar treated in both normal and IL-1 *β*-stimulated explant conditions performed rather efficiently, and the treatments with FQs were successful in enhancing the proteoglycan content in the matrix of the explant cultures in the pre-IL-1 *β*-stimulated explant conditions at T3 treatment. At T5, the safranin-O staining in a single Enro treatment group (Figs. 5C, 5I and  5M) was stronger than that in the other groups in every explant condition, particularly in the IL-1 *β*-stimulated explants (Figs. 5I and  5M). The decrease of proteoglycan staining was significantly found in HA combined with Enro treatment in every explant condition, and the major decrease in intensity was found in the non + HA + Enro group (decrease to 91.60%) and was lower than that in the non + Enro treatment (*P* < 0.0001; Fig. 5U) .

### Effects of FQs with and without HA on articular cartilage genes

**Figure 6:**
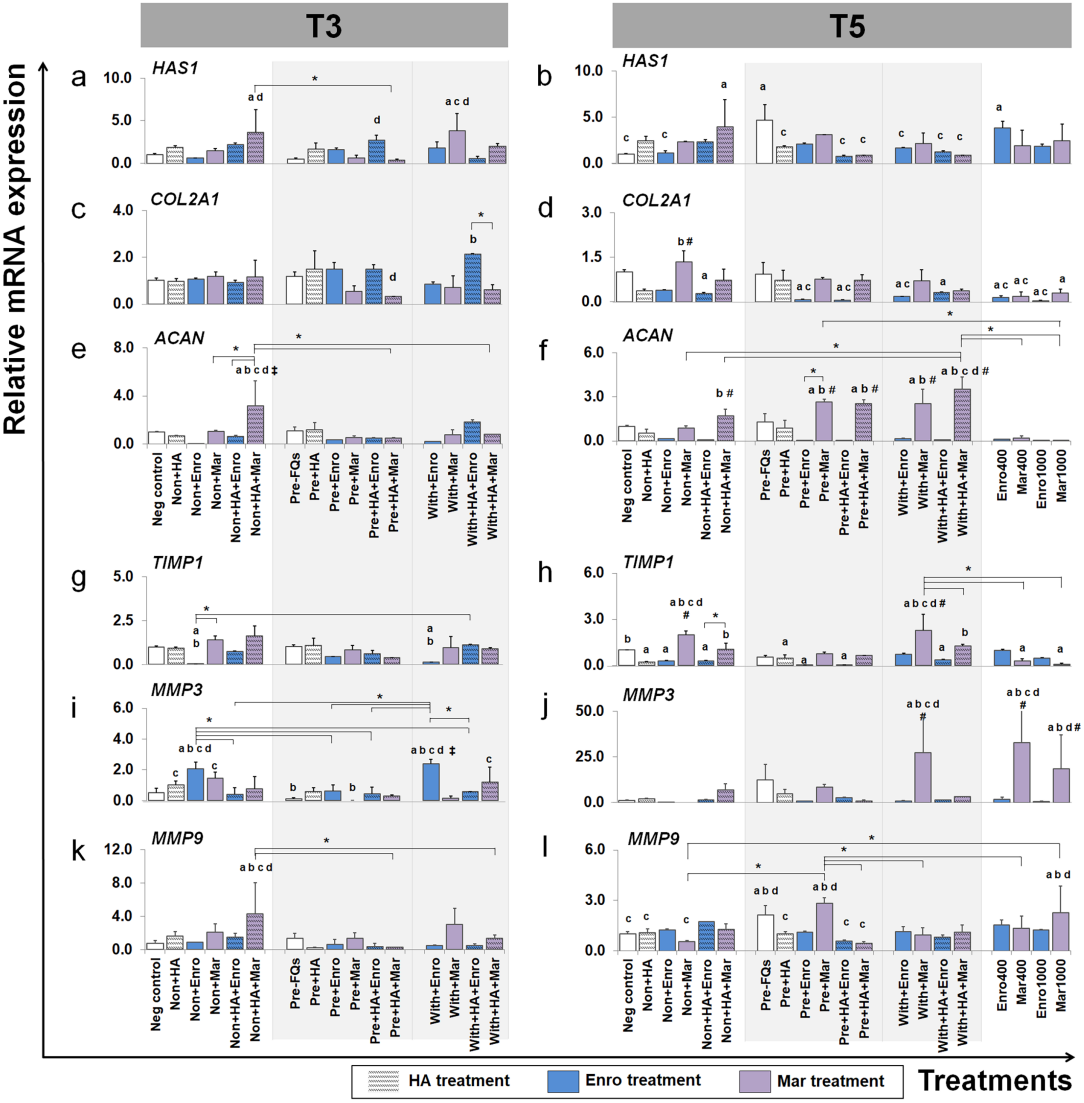
Effects of FQs with and without HA on ECM genes and protease genes on canine cartilage explants. The expression levels of ECM component genes, *HAS1, COL2A1* and *ACAN,* as well as genes involved in the catabolic process, *MMP3, MMP9* and their inhibitor *TIMP1*, in conditioned cartilage explants harvested at T3 and T5 are presented as insets (A–H). Relative gene expression was normalized to the mean housekeeping gene expression and the negative control group. The dark blue bars denote Enro treatment groups and the light-pastel purple bars denote Mar treatment groups. Data are presented as the mean ±  SD (*n* = 3). Significant differences are considered to exist when *P* < 0.05: “a” represents significant differences compared with the negative control group, “b” represents significant differences compared with the non + HA group, “c” represents significant differences compared with the pre-IL-1b induction group (pre, −FQs), “d” represents significant differences compared with the pre + HA group, # represents significant differences compared with the Enro-treated groups, ‡ represents significant differences compared with the Mar-treated groups, and asterisk (*) denotes significant differences between the treatment groups.

**Figure 7:**
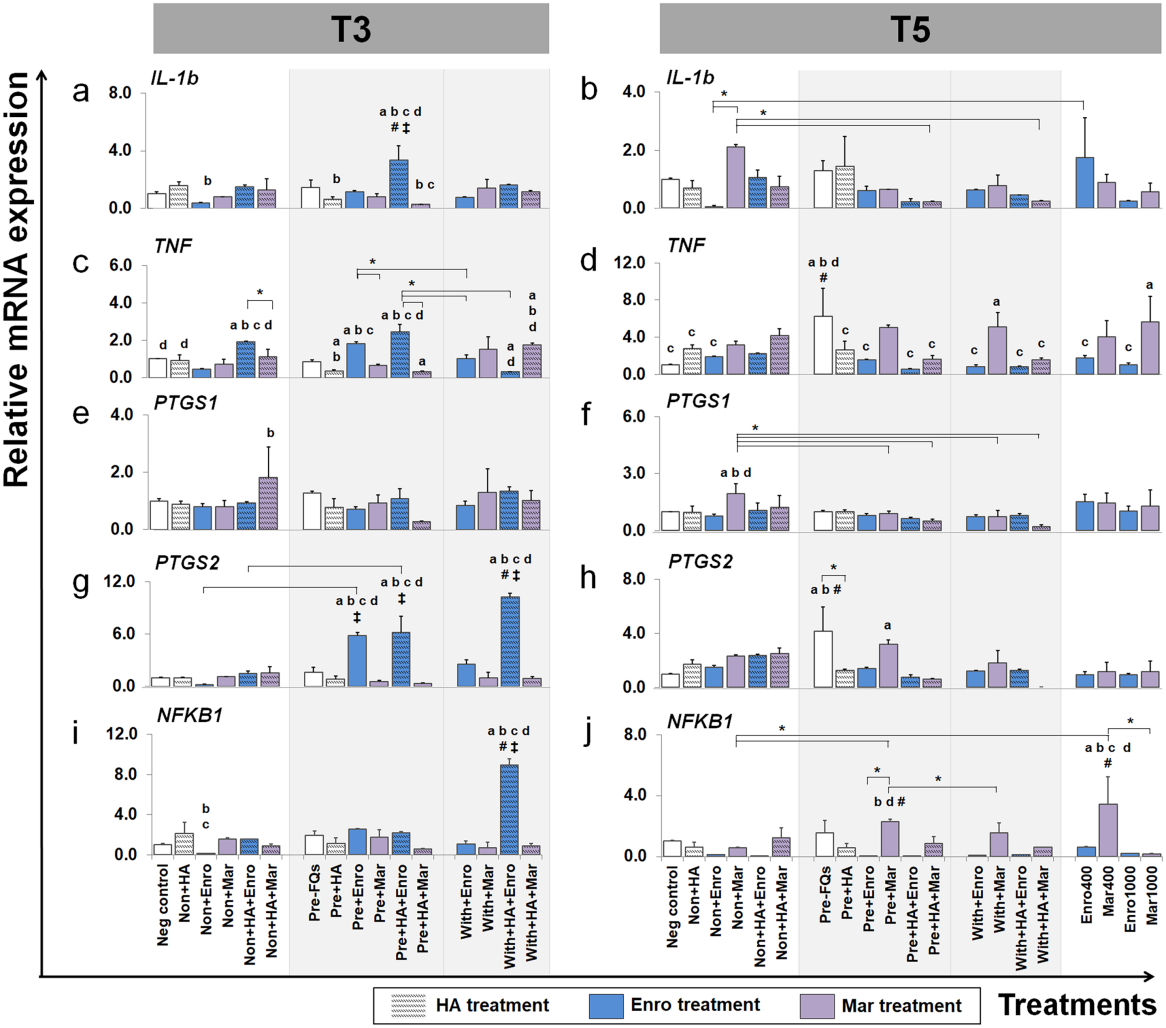
Effects of FQs with and without HA on the key cytokines and enzymes expression. This group of gene were a key cytokines and enzymes involve in inflammatory pathway of cartilage. The expression levels of *IL-1b*, *TNF*, *PTGS1*, *PTGS2* and *NFKB1* in conditioned cartilage explants harvested at T3 and T5 are presented as insets (A–J). Relative gene expression was normalized to the mean housekeeping gene expression and the negative control group. The dark blue bars denote Enro treatment groups and the light-pastel purple bars denote Mar treatment groups. Data are presented as the mean ± SD (*n* = 3). Significant differences are considered to exist when *P* < 0.05: “a” represents significant differences compared with the negative control group, “b” represents significant differences compared with the non + HA group, “c” represents significant differences compared with the pre-IL-1b induction group (pre, −FQs), “d” represents significant differences compared with the pre + HA group, # represents significant differences compared with the Enro-treated groups, ‡ represents significant differences compared with the Mar-treated groups, and asterisk (*) denotes significant differences between the treatment groups.

The effects of FQs on mRNA expression in conditioned explants of some selected genes that code for cytokines and enzymes that respond to inflammatory pathways and the ECM component are shown in Figs. 6 and 7.

### Effects of FQs on ECM component genes

We examined which anabolic pathways of conditioned cartilage disks regulate at different days of cultivation (T3 and T5). At T3, *HAS1* was highly expressed in non + HA + Mar (*P* = 0.014) and with + Mar (*P* = 0.010) compared with the negative control. The most significant increases in expression of *COL2A1* were found in the with + HA + Enro treatments groups, which were up-regulated higher than the normal explant treated with HA alone (non + HA). The most significant increase in *ACAN* expression was found in only one treatment “non + HA + Mar,” which was significant in all Mar treatment and most of Enro treatments. At T5, *HAS1* was highly expressed in the pre-IL-1 *β*-stimulated explant (pre, −FQs) group, non + HA + Mar, and positive control (Enro 400) compared with the negative control. Treatment with HA significantly reduced *HAS1* regulation in the IL-1 *β*-stimulated explants, lower than the pre, −FQ group (close to the negative control group). Treatment of the explants with Enro affected *COL2A1* expression, whereas Mar-treated groups did not cause any changes (no statistically significant differences compared with the negative control). Most significant increases in *ACAN* expression were found in Mar treatment groups and were highly expressed when in combination treatment with HA. However, a high dose of both FQ (at 400 and 1,000 µg/µL) treatments activated the explants down-regulation in the *COL2A1* and *ACAN* expressions.

### Effects of FQs on the expression of matrix metalloproteinase and their inhibitor

*TIMP1* was significantly down-regulated in the Enro treatment groups at both T3 and T5, particularly in the IL-1 *β*-stimulated explants (both pre and with) compared with negative control. Treatments of non + Enro and with + Enro at T3 were found to significantly up-regulate *MMP3* compared with all Enro-treated and control groups. At T5, Mar induced *TIMP1* up-regulation higher than other treatments of Enro, particularly in the non + Mar and with + Mar groups compared with the negative control (*P* = 0.002 and *P* = 0.00026, respectively). The same trend was found in the *MMP3* gene at T5, which had very low expression levels in the Enro treatment groups but highly expressed in Mar treatment. The most significant increases in *MMP3* expression were found in high concentrations of Mar treatment at 400 µg/mL (positive control) up to 33-fold. Treatment of FQs on IL-1 *β*-stimulated explants regulated the down-expression of *MMP9* at T3, except in the non + HA + Mar group at T3, which exhibited higher expression levels compared with all Enro-treated and control groups. At T5, the pre-IL-1 *β*-stimulated (pre, −FQs), pre + Mar, and Mar 1,000 groups were found to significantly up-regulate the *MMP9* gene compared with the negative control group (*P* = 0.02, *P* = 0.003, and *P* = 0.04, respectively). Moreover, HA co-treatment successfully reduced *MMP9* gene expression between pre + Mar and pre + HA + Mar (*P* = 0.001).

### Effects of FQs on pro-inflammatory cytokines *IL-1b* and *TNF*

FQ treatments induced *IL-1b* and *TNF* expression up-regulation in both normal and IL-1 *β*-stimulated explant conditions and at different time periods. The expression level of *IL-1b* after treatment (at T3) was distinctively up-regulated in the pre + HA + Enro group, comparing between all treatment conditions (*P* ≤ 0.05). Moreover, Enro induced *TNF* expression up-regulation in normal and pre-IL-1 *β*-stimulated explants, pre + HA + Enro had a significantly higher expression compared with other groups, and the most significant was found when compared with pre + HA (*P* < 0.0001). The highest expression level of *IL-1b* at T5 was found in the non + Mar group; however, statistically significant differences were found when compared with the negative control. The expression levels of *TNF* switched to up-regulation in Mar-treated groups compared with Enro-treated and control groups at T5, and the expression levels were significantly increased in the with + Mar and positive control groups (Mar 1000), although the highest expression level was found in the pre, −FQs group.

### Effects of FQs on *PTGS1*, *PTGS2*, and *NFKB1*

A slight difference was found in the expression pattern of the *PTGS1* gene at T3 and T5, although treatment with Mar in normal explants (non + HA + Mar at T3 and non + Mar at T5) exhibited higher expression levels compared with all Mar-treated and control groups. Contrary effects were found for treatment with FQs for *PTGS2* and *NFKB1* genes at a different time period. At T3, the *PTGS2* gene extremely up-regulated in the Enro-treated groups compared with all Mar-treated groups and the control group, with the highest expression level in the with + HA + Enro group (*P* < 0.0001) compared with all treatment groups and the negative control. Also, the *NFKB1* gene significantly exhibited higher expression levels in the with + HA + Enro group compared with all treatment groups and the control group (*P* < 0.0001). However, at T5, the expression levels of both *PTGS2* and *NFKB1* in Enro-treated groups sharply decreased, to levels similar or lower than control groups. Whereas, the pre + Mar and the Mar 400 groups were there significant induced *NFKB1* expression up-regulation compared with all of Mar treatment groups and the control group.

## Discussion

This study used canine cartilage explants to examine and compare the direct effects of Enro and Mar with both the absence and presence of HA. Cartilage explants were pre-induced with 20 ng/mL of IL-1 *β* for 48 h, which sufficiently induced the inflammation model in bovine articular cartilage explants (Badger et al., 1998; Torzilli, Bhargava & Chen, 2011) and activated an inflammatory response, but did not have any cytotoxic effect on chondrocyte cultures (Siengdee et al., 2016). Due to the presence of IL-1 *β* pre-induction at 48 h, the conditioned cartilage explants were attended to every 48 h cycle, but not longer than 14 days as the cartilage explants would begin to lose ECM content and chondrocyte viability (Pallante et al., 2009). Here, different durations of treatment at T3 and T5 (days 4 and 8 after treatment, respectively) represented short- and long-term changes from a single dose of FQ and HA as has been described previously (Siengdee et al., 2016).

Our results suggested the following:

1. Mar treatment significantly decreased UA content and directed GAG degradation in normal explants. However, HA combined with Mar-treated conditions performed rather efficiently and successfully enhanced the UA content in normal explants and GAG content in the matrix of both normal and pre-IL-1 *β*-stimulated explants in a short-term period.
2. Enro treatment effectively stimulated proteoglycan anabolism by increasing the UA content and GAGs in normal and pre-IL-1 *β*-stimulated explants, but co-treatment with HA and Enro reduced the GAG content in the matrix of both normal and pre-IL-1 *β*-stimulated explants in a long-term period.
3. Combined HA and both FQs increased s-GAG release. By contrast, it significantly increased the UA content remaining in the normal explants and down-regulated the expression of *MMP3* at both short- and long-term periods.
4. FQs specially promote gene expression involved in matrix production, pro-inflammatory cytokines, and cell degradation, depending on short- and long-term durations.
5. Combined HA and Mar effectively up-regulated *HAS1* and *ACAN* expressions and also reduced the expression of *MMP9*, *TNF*, *PTGS2*, and *NFKB1* at long-term durations, suggesting the beneficial effects of HA in reducing the adverse effects of FQs, particularly in Mar treatment at the transcriptional level.

DMMB binding assay, carbazole colorimetric assay, and safranin-O staining are the techniques used to optimize the GAG content. This study ensures that the results are as reliable as possible. DMMB binding assay was suitable in measuring the type of s-GAG, excluding HA, keratan sulfates, which lack UA and could not be detected using carbazole colorimetric assay (Ulf et al., 2017).

No significant difference of cumulative s-GAG release was found between Mar and Enro treatments alone in our model. These patterns of cumulative s-GAG release in IL-1 *β*-induced groups were found at T2 and T3 (after pre-induced for 48 h) rather than depending on our IL-1 *β* simulation period. However, in this condition, combined FQs and HA might be related to s-GAG release, which confirmed our previous report that co-culturing Enro with HA could modify s-GAG synthesis (Siengdee et al., 2016). Interestingly, s-GAG level was reversibly adjusted at T5 after removing all drug treatments, which may reflect a recovery process compared with long-term FQ treatments in the positive control groups, which still had high s-GAG level at T5. Some reports also described the repair-like processes after FQ treatment (Kato & Onodera, 1988) and the changes of sulfate reversibility after 48 h of a single administration of FQ (Simonin et al., 1999).

The UA content and safranin-O intensity results were consistent, suggesting that a single treatment at 200 ng/mL of Enro may also stimulate proteoglycan anabolism in this model. By contrast, treatment with Mar *resulted in the opposite*. However, data of Mar on cartilage are still limited, and its effects have not been sufficiently investigated, whereas the effects of Enro have been abundantly reported, such as decreasing the matrix components, such as collagens and proteoglycans, and decreasing the total monosaccharide content of the chondrocytes and tendon cells *in vitro* and *in vivo* of various species, such as sheep (Khazaeel et al., 2015; Khazaeil et al., 2012) horse (Yoon et al., 2004a; Yoon et al., 2004b), and dog (Lim et al., 2008). The combination of HA and FQs increased the UA content and safranin-O intensity in normal explants at both short and long terms. This both reflected the GAG and proteoglycan contents remaining in the cartilage explants (Schmitz et al., 2010) and may suggest the metabolically active chondrocytes after drug treatment (Shepard & Mitchell, 1976).

Pre-induced cartilage explants with IL-1 *β* mimicked the initial inflammatory environment from joint infection or OA. As expected, the expression levels of *HAS1*, *MMP9*, *TNF*, and *PTGS2* were abundantly up-regulated after IL-1 *β* induction, which increased the catabolic process in the explants and may produce different responses after drug treatment (Dozin et al., 2002; Sun, Wu & Weng, 2015). Our findings indicated that Enro and Mar both affect inflammatory mediators and ECM component-encoding genes. Enro induced IL-1 *β*-stimulated explants to up-regulate *MMP3*, *TNF*, and *PTGS2* genes and reduced long-term *TIMP1* expression, whereas ECM coding gene expression was inhibited for a long term. Although Mar caused higher expressions of *ACAN* and *TIMP1*, it also induced cartilage explants to overexpress *MMP9*, *TNF*, *PTGS2*, and *NFKB1* for a long term. The results presented in this paper have confirmed our previous data in canine chondrocytes, which showed that Enro-regulated inflammation-induced cells overexpress *TNF* and *MMP3*, whereas Mar induced up-regulation of *PTGS2*, *NFKB1*, and matrix metalloproteinases and their tissue inhibitor *MMP9* and *TIMP1* and also enhanced the expression of ECM component genes *HAS1*, *COL2A1*, and *ACAN* (Siengdee et al., 2016).

With regard to the effects of FQs on articular cartilage genes, we suggest that both FQs may selectively stimulate conditioned explants to express MMP genes and may alter pro-inflammatory genes and ECM component genes in different directions. This might result in an unbalance between the expression of catabolic and anabolic factors in the cartilage similar to early-stage OA (Kuyinu et al., 2016). Similar to our findings, FQs have been previously reported to selectively increase MMP family expression in the tendon cells (Corps et al., 2002; Corps et al., 2005; Sendzik et al., 2005). FQs also induce inflammatory mediators or cytokines, e.g., *TNF α,* interleukin-8, *IL-8* (Nakajima et al., 2018), and apoptosis marker (Sendzik et al., 2005). The influence of the chemical structure between Enro and Mar has been discussed earlier (Siengdee et al., 2016). We suggest that the severity of the effect is less toxic than methylpiperadinyl (in Mar structure) in cartilage degradation based on the chemical structure of two substituents at R-1 and R-7, ethylpiperadinyl substituent at R-7 of the FQ ring (in Enro structure). The cyclopropyl substituent at R-1 in Enro structure has a suppressive effect on matrix production, e.g., collagen and integrin, as ciprofloxacin (Sendzik et al., 2005).

In this study, simultaneous treatment of HA was aimed to use the chondroprotective effect of HA (Bruyère et al., 2016; Maheu, Rannou & Reginster, 2016) to reduce the adverse effects of FQ treatment. HA size (MW ∼500–730 kDa) in this study was categorized as the medium molecular weight or MMW-HA (Monslow, Govindaraju & Puré, 2015), which has been approved for use in clinical applications (Goldberg & Buckwalter, 2005). Based on the specific properties that exist in this molecular weight range (500–1,000 kDa), HA can penetrate through synovial tissue to promote synovial fibroblasts to synthesize new molecules of HA (*in vivo*) (Coleman et al., 2000; Ghosh, 2002). In this study, MMW-HA itself seemed to not strongly affect chondrocyte function at the transcription level. However, it is assumed that HA might be able to adhere to the explant tissue surface and create a matrix together with its viscous property which could protect the underlying tissue from exposure to these drugs and IL-1 *β*, along with the resulting tissue homeostasis. This would ultimately reduce ECM loss and could enhance proteoglycan anabolism. Combination of HA with other drugs was successful in reducing their cytotoxic effects, such as when combined with corticosteroids (Bannuru et al., 2009) and non-steroidal and anti-inflammatory drugs (Euppayo et al., 2015). Our findings showed that co-treatment with HA and FQs can possibly reduce the negative effects of FQs on conditioned canine explants, particularly in the case of HA combined with Mar, which was contrary to our previous studies in canine chondrocytes (Siengdee et al., 2016). This study showed that combination of HA with Mar can activate both normal and IL-1 *β*-stimulated explants to increase the expression of keys of ECM, i.e., *HAS1* and *ACAN*, in normal condition, down-regulation of *TIMP1*, matrix-destructive enzyme inhibitors, down-regulation of critical matrix-degrading enzyme *MMPs*, and down-regulation of *IL-1b*, *TNF*, *PTGS2*, and *NFKB1* in IL-1 *β*-stimulated explants. These results suggest the long-term protective effect of HA in canine cartilage explants. By contrast, combination of HA with Enro successfully decreased the expression level of *MMP3* and increased the expression level of *TIMP1* in normal conditions but caused an extensive destruction by increasing the expression of *IL-1b*, *TNF*, *PTGS2*, and *NFKB1* in IL-1 *β*-stimulated explants for a long term. This phenomenon confirmed the possible beneficial effect of HA in reducing the adverse effects of FQ treatment at the transcriptional level in cartilage explants.

The present findings were obtained from an *in vitro* study which certainly had some limitations. Based on previous literature (Badger et al., 1998; Torzilli, Bhargava & Chen, 2011; Siengdee et al., 2016), inflammatory stimulation was induced at 48 h and drug treatments were only investigated every 48 h until the conditioned explants were collected on day 8. The limited drug exposure duration in these culture systems may result in limitations in the determination of the HA-treatment effects. There also could be differences in molecular responses due to the *in vitro* vs. *in vivo* model systems. Notably, the findings may not be relevant to long-term *in vivo* responses, which can vary from 1 up to 16 weeks (Nganvongpanit et al., 2013; Pashuck et al., 2016; Wanstrath et al., 2016). This study used only IL-1 *β* that was known to have a major pro-inflammatory cytokine response in inflammatory arthritis (Johnson, Argyle & Clements, 2016). This function mimics that of an articular inflammation model, but may be insufficient as a representative of the complexity of the inflammatory process *in vivo*. Moreover, drug treatments sometimes differ when comparing results between *in vitro* and *in vivo* systems, the latter of which involves more complex interactions between adjacent tissues of the joints (Johnson, Argyle & Clements, 2016; Sokolove & Lepus, 2013; Tarafder & Lee, 2016). Furthermore, there is limited empirical evidence showing how HA acts against the toxicity of drugs. Up to this point, we have no information that would enable us to explain or even speculate on the possible mechanisms or interactions between HA and FQs. Clearly, further studies should address these current limitations by applying more complex test set-ups and measurements. Both in vivo and in vitro platforms are needed to investigate the relationship between the chemical structure of FQs and the severity of cartilage damage. To date neither can confirm the potential interaction between their chemical structures in combination with HA at the pharmacodynamic and pharmacokinetic levels. The determination of which may help to reveal comprehensive information, thus giving veterinarians the option to control the joint infection and effectively limit OA progression.

## Conclusion

We have demonstrated the direct effects of Enro and Mar on canine cartilage explants in both normal and interleukin-1 beta (IL-1 *β*)-stimulated conditions. Our findings suggest that both FQs may alter the proteoglycan anabolism and selectively stimulate the conditioned explants to express MMP-encoding genes, inflammatory mediators, and ECM components in different directions, that might further cause some articular cartilage lesions. Therefore, the use of FQs may possibly increase the risk of articular cartilage degradation. Controlling the joint infection along with limiting OA progression are critical; therefore, these factors should be taken into consideration before medication is given. In this study, HA was effective in reducing the adverse effects of FQs by restoring s-GAG levels, while maintaining the UA content and down-regulating the expression of *MMP3*. Combined, HA and Mar effectively up-regulated *HAS1* and *ACAN* expressions and also reduced the expression levels of *MMP9, TNF, PTGS2,* and *NFKB1* at long-term durations. However, further research in the *in vivo* model is needed to confirm the effects of these FQs and the interaction between their chemical structures in combination with HA.

##  Supplemental Information

10.7717/peerj.6553/supp-1 Supplemental Information 1 Raw data

## Additional Information and Declarations

### Data Availability

The following information was supplied regarding data availability: The raw measurements are available in Supplemental File.
